# Controlling the Orientation of MoS_2_ Films on Mo Metal Thin Film Through Sulfur Flux Regulation: A Novel Reaction-Diffusion Model

**DOI:** 10.3390/nano15231783

**Published:** 2025-11-27

**Authors:** Joonam Kim, Masakazu Ike, Kenichi Tokuda

**Affiliations:** National Agriculture and Food Research Organization, Research Center for Agricultural Department Robotics, Kannondai 1-31-1, Tsukuba 305-0856, Japan

**Keywords:** MoS_2_, orientation control, metal thick film, sulfur source, bubbler, vertical vs. horizontal

## Abstract

This study presents a novel strategy for controlling the orientation of MoS_2_ films on thick metallic substrates through precise regulation of the sulfur flux alone. In contrast to previous approaches that rely on substrate modifications or complex parameter tuning, orientation control is achieved here solely by adjusting the sulfur concentration during the sulfurization of 400 nm RF-sputtered Mo films. The metallic Mo substrate also allows potential film transfer via selective etching—analogous to the graphene/Cu system—providing a viable route for device integration on arbitrary substrates. Analyses (XRD, Raman, and TEM) reveal that low sulfur flux (30–50 sccm) favors horizontal growth, whereas high flux (>300 sccm) induces vertical orientation. To rationalize this behavior, a reaction-diffusion model based on the Thiele modulus was developed, quantitatively linking sulfur flux to film orientation and identifying critical thresholds (~50 and ~300 sccm) governing the horizontal-to-vertical transition. This unified approach enables the realization of distinct MoS_2_ orientations using identical materials and processes, analogous to the orientation control in graphene growth on copper. The ability to grow orientation-controlled MoS_2_ on non-noble metal substrates opens new opportunities for integrating electronic (horizontal) and catalytic (vertical) functionalities, thereby advancing scalable manufacturing of TMDC-based technologies.

## 1. Introduction

Two-dimensional (2D) materials with sub-nanometer thickness have garnered significant attention owing to their distinctive physical and chemical properties at the nanoscale [1]. The commercialization of graphene—the first widely recognized 2D material—marked a pivotal milestone in this field [2,3]. This breakthrough largely stemmed from the development of scalable graphene growth techniques on Cu substrates, a non-noble metal that supports roll-to-roll manufacturing and optimized chemical vapor deposition (CVD) processes [4,5,6]. Such advancements in substrate engineering transformed graphene from a laboratory curiosity into a commercially viable material, enabling diverse applications spanning electronic devices such as transistors [7], sensors [8], and optoelectronics [9,10], to energy systems including solar cells [11], fuel cells [12], and energy harvesters [13], as well as energy storage devices [14,15,16] and flexible electronics [17]. The ability to synthesize large-area, high-quality graphene films on affordable and readily available substrates such as Cu has been instrumental in overcoming the limitations of conventional graphene production methods [18,19]. The widespread success of graphene underscores a central principle in 2D materials research [20]: growth on non-noble metal substrates provides key advantages over single-crystal counterparts, including simplified processing, cost-effectiveness, and facile transfer to application-specific substrates [21,22].

While graphene growth methodologies have been extensively optimized, the synthesis of other promising 2D materials—particularly transition metal dichalcogenides (TMDCs) such as molybdenum disulfide (MoS_2_)—remains largely restricted to noble-metal substrates such as gold [22,23] or single-crystal substrates with limited surface reactivity [24,25,26,27,28,29]. Recent progress in MoS_2_ growth includes innovative techniques such as isolated plasma soft deposition for large-area 2D film formation [30] and electrochemical deposition methods that allow precise control of surface properties [31]. Despite their excellent intrinsic properties, the commercialization and widespread adoption of these materials remain constrained by such substrate and process limitations. Among TMDCs, MoS_2_ stands out owing to its tunable electronic characteristics, high surface area, and mechanical flexibility, making it highly attractive for diverse applications: catalysis [32], energy storage [33,34], optoelectronics [35], and sensing [36,37]. Recent comprehensive reviews have highlighted MoS_2_’s expanding role in advanced energy storage systems [38,39], its remarkable potential for biosensing applications [40], and its exceptional catalytic performance with edge-site activation for hydrogen evolution reactions [41,42].

Interestingly, the crystallographic orientation of MoS_2_ nanosheets relative to the substrate critically determines their physical and chemical properties, enabling orientation-specific functionality. Vertically oriented MoS_2_ exposes a high density of catalytically active edge sites, making them particularly effective for electrochemical applications such as hydrogen evolution reaction (HER) catalysis [40,41,42]. In contrast, horizontally oriented MoS_2_ nanosheets exhibit excellent in-plane charge-transport characteristics along the basal plane, rendering them highly suitable for electronic and optoelectronic devices. Conventional methods for controlling MoS_2_ nanosheet orientation typically rely on entirely distinct synthesis routes for each desired alignment. Vertically oriented MoS_2_ is usually obtained by high-concentration sulfurization of Mo films [43,44,45,46], whereas horizontally oriented MoS_2_ requires precisely controlled CVD growth on single-crystal or noble-metal substrates, or the sulfurization of ultrathin (<3 nm) Mo films [47,48]. These disparate methodologies inherently increase process complexity and hinder the integration of both orientations within a unified device architecture. Recent studies—such as step-engineered nucleation and domain orientation control in WSe_2_ epitaxy [49]—highlight the broader importance of understanding orientation mechanisms in TMDC growth. Likewise, Sojková et al. demonstrated orientation tuning in ultrathin (~3 nm) Mo films via heating-rate modulation [47], emphasizing the sensitivity of film alignment to growth kinetics.

This study fundamentally extends the current understanding of orientation control in TMDC growth by demonstrating, for the first time, that the orientation of MoS_2_ nanosheets on relatively thick (400 nm) RF-sputtered Mo films can be precisely regulated solely through sulfur flux modulation during sulfurization. Unlike previous studies that relied predominantly on empirical observations, this work establishes a quantitative reaction-diffusion framework based on the Thiele modulus to elucidate the orientation-transition mechanism and identify the critical sulfur-flux thresholds governing the growth-regime transitions. This represents a significant breakthrough in TMDC synthesis: the ability to control MoS_2_ orientation on non-noble metallic substrates mirrors the transformative role of Cu in scalable graphene growth, which was pivotal to its commercialization. The proposed strategy offers a unified and versatile route to synthesize horizontally and vertically oriented MoS_2_ using identical materials and processing conditions, thereby introducing a new paradigm for crystallographic structure control in 2D materials. Moreover, the use of a metallic Mo substrate presents a practical advantage—the potential for film transfer via selective chemical etching, analogous to graphene transfer from Cu foils. MoS_2_ films grown on Mo can thus be transferred to target substrates using etchants such as (NH_4_)_2_S_2_O_8_ or FeCl_3_, facilitating integration with diverse device platforms [50,51].

Comprehensive experimental characterization using transmission electron microscopy (TEM), Raman spectroscopy, and X-ray diffraction (XRD) was performed to investigate the orientation-transition phenomenon in MoS_2_ films under varying sulfur flux. To interpret these results, a reaction-diffusion theoretical framework based on the Thiele modulus was developed, quantitatively explaining the physical mechanism underlying orientation control through the competition between sulfur surface reaction and diffusion into the film. Although temperature and Mo film thickness are established factors in MoS_2_ growth [43,45], this study deliberately isolates sulfur flux as the primary variable to demonstrate its independent influence on film orientation. The Mo film thickness of 400 nm was selected based on previous studies showing typical vertical MoS_2_ growth on thick metallic substrates in this thickness range [43]. This thickness represents a sufficiently thick metallic substrate where surface growth dynamics are decoupled from substrate thickness effects, making it representative of practical device-relevant conditions. Although film thickness is known to affect crystalline quality [45], this study deliberately focuses on orientation control—which is governed primarily by sulfur flux at the growth interface—rather than comprehensive thickness optimization. Beyond elucidating TMDC growth mechanisms, this study presents a scalable and controllable approach for growing orientation-controlled MoS_2_ on non-noble metal substrates, offering a pathway toward commercialization of MoS_2_-based technologies. The demonstrated ability to precisely control orientation on practical substrates opens new possibilities for integrated device architectures requiring both vertical (catalytic) and horizontal (electronic) functionalities, potentially advancing industrial applications of 2D materials.

## 2. Materials and Methods

In this study, 400 nm-thick Mo films were deposited on piranha-solution-treated SiO_2_/Si substrates via RF sputtering at room temperature. Partial sulfurization of these thick Mo films was intentionally employed to investigate the mechanisms governing orientation control on metallic substrates. The underlying Mo layer acts as a conductive metallic base, analogous to bulk Si beneath the surface oxide layer in silicon wafers. Complete sulfurization was neither intended nor required, as orientation control is primarily determined by sulfur flux at the growth interface rather than by full film conversion.

**Figure 1:**
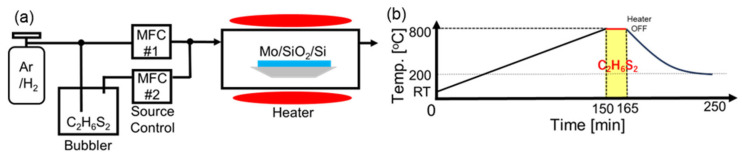
(**a**) Schematics of the CVD setup used for MoS_2_ growth. (**b**) Temperature profile used in the synthesis process.

The experimental setup for MoS_2_ synthesis is shown in Figure 1a. Here, CVD was carried out at atmospheric pressure using dimethyl disulfide (C_2_H_6_S_2_) as the sulfur precursor, supplied through a bubbling system. This precursor was chosen instead of conventional sulfur powder because elemental sulfur exhibits multiple crystalline phases with temperature-dependent evaporation behavior, making low-concentration control difficult [52,53]. In contrast, C_2_H_6_S_2_ enables a wide and precisely tunable range of sulfur concentrations [54]. Sulfur flux was accurately controlled using a dual mass-flow controller (MFC) system: MFC #2 regulated the C_2_H_6_S_2_ precursor flow, while MFC #1 controlled the Ar/H_2_ carrier gas (Figure 1a). The Ar/H_2_ carrier gas was supplied from a pre-mixed cylinder with a fixed composition ratio of 96:4. The total gas flow was fixed at 1000 sccm, and the relative flow ratio between MFC #1 and MFC #2 was adjusted to modulate the sulfur concentration in the reaction chamber. This configuration allowed fine sulfur flux tuning, enabling precise control of the MoS_2_ nanosheet orientation.

The temperature profile employed in this study is illustrated in Figure 1b. The substrate was heated from room temperature to the growth temperature of 800 °C at a controlled rate of 5 °C/min. This relatively slow ramp rate was chosen to minimize thermal stress in the Mo thin film, thereby preventing delamination from the SiO_2_/Si substrate and promoting the growth of larger Mo crystalline domains. As reported by Stern et al. [43], controlled heating can suppress the formation of randomly oriented MoS_2_ layers that often arise during rapid temperature increases, improving the overall film crystallinity. After reaching 800 °C, the sulfur precursor was introduced into the chamber, and MoS_2_ growth proceeded for 15 min. The system was then allowed to cool naturally to 200 °C with the heater turned off, following an exponential decay profile, after which the chamber was opened to accelerate cooling to room temperature. This approach balances thermal stress minimization with experimental efficiency.

**Figure 3:**
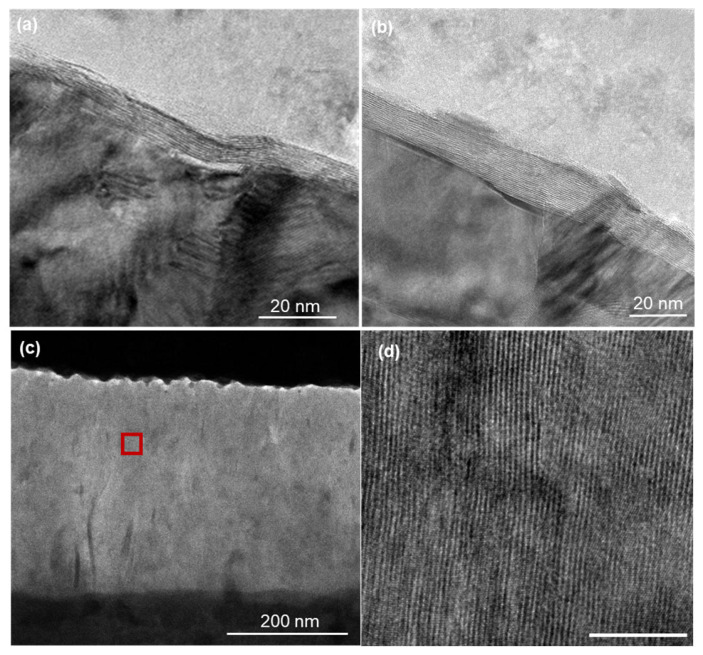
Cross-sectional TEM images of MoS_2_ films showing (**a**,**b**) horizontally aligned MoS_2_ layers grown under a sulfur flow rate of 30 sccm, (**c**) overall structure of vertically aligned MoS_2_ film grown under 1000 sccm, and (**d**) magnified view of the region indicated by the red square in (**c**), showing the vertical layer alignment in detail.

The growth duration of 15 min was selected based on preliminary experiments to ensure sufficient MoS_2_ formation for reliable XRD and Raman characterization while maintaining clear orientation transitions. Cross- sectional TEM analysis of the sample grown at 1000 sccm (Figure 3c) revealed that approximately 200 nm of the Mo film was converted to form ~350 nm of MoS_2_, with ~200 nm of unreacted Mo remaining at the substrate interface. This partial sulfurization was intentional, as complete conversion of the 400 nm Mo film was neither necessary nor desired for investigating orientation control mechanisms. The retained metallic Mo layer serves both as a growth template and as a potential sacrificial layer for future film transfer applications analogous to the graphene/Cu system [50,51].

Statistical analysis was conducted using at least three independent samples for each sulfur flow rate condition. The threshold transitions observed at approximately 50 sccm and 300 sccm were consistently reproduced across multiple growth batches, with standard deviations within ±10 sccm for the critical thresholds.

XRD measurements were performed using Cu Kα radiation (λ = 1.54 Å) over a 2θ range of 10–60°, with a step size of 0.02° at room temperature. Raman spectra were acquired using a 532 nm excitation laser with a spectral resolution of 1 cm^−1^ and a laser power of 10 mW to minimize thermal effects. For each sample, spectra were collected from at least five distinct locations to ensure spatial representativeness. Cross-sectional transmission electron microscopy (TEM) specimens were prepared using focused ion beam (FIB) milling, and high-resolution TEM imaging was conducted at an acceleration voltage of 200 kV. Each data point represents the average of measurements taken from at least three different regions per sample to account for spatial variability. The reproducibility of the observed threshold transitions was validated across multiple independent growth experiments conducted under identical conditions.

## 3. Results

**Figure 2:**
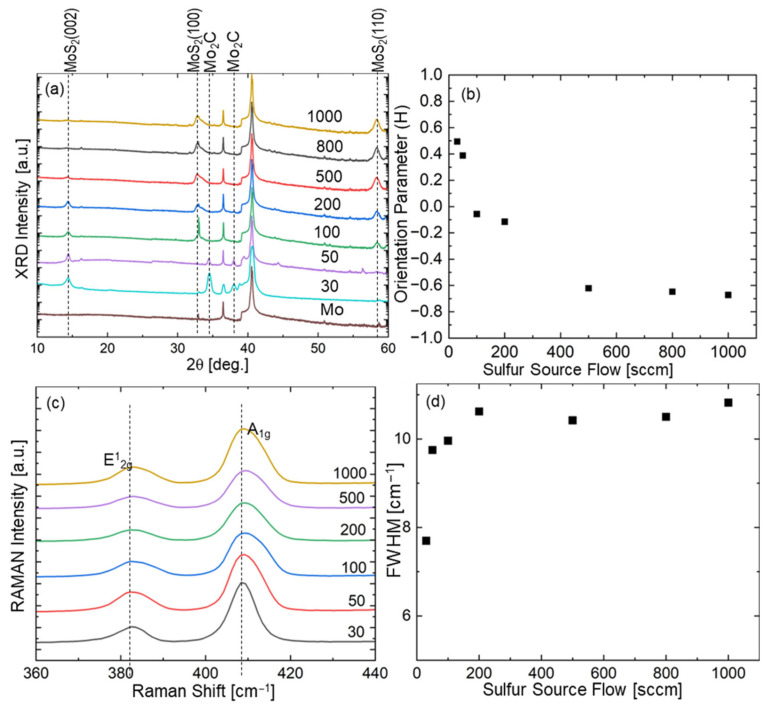
(**a**) XRD patterns of MoS_2_ films grown with varying sulfur flow rates (representative data from n = 3 samples per condition). (**b**) Orientation parameter (*H*) derived from XRD peak intensity ratios, defined as *H* = (*A* − *B*)/(*A* + *B*), where *A* and *B* represent the normalized intensities of the MoS_2_ (002) peak (horizontal orientation) and MoS_2_ (110) peak (vertical orientation), respectively. (**c**) Raman spectra showing the characteristic *E*^1^_2g_ and *A*_1g_ modes. (**d**) Full width at half maximum (FWHM) of the *A*_1g_ peaks as a function of sulfur flow rate.

The crystallinity and orientation of MoS_2_ films as a function of sulfur flow rate were systematically evaluated using XRD and Raman spectroscopy (Figure 2). The XRD patterns in Figure 2a reveal the evolution of crystal orientation with increasing sulfur flux. At the highest sulfur flow rate of 1000 sccm, the diffraction pattern exhibits a pronounced MoS_2_ (110) peak, indicative of vertically aligned MoS_2_ (V-MoS_2_) layers [36,40]. This observation aligns with previous reports of V-MoS_2_ thin films typically grown under high sulfur-density conditions via sulfur powder evaporation. A minor MoS_2_ (002) peak is also present at 1000 sccm, likely corresponding to a randomly oriented layer formed during the initial stages of vertical growth [40].

As the sulfur flow rate is systematically reduced, the growth orientation transitions from vertical to horizontal: the intensity of the MoS_2_ (110) peak, associated with vertical growth, gradually decreases, while that of the MoS_2_ (002) peak, indicative of horizontal layering, correspondingly increases [47,48,55,56]. At the lowest flow rate of 30 sccm, the XRD pattern shows predominantly horizontal orientation, with a strong MoS_2_ (002) peak and an almost undetectable MoS_2_ (110) signal. Notably, the XRD patterns of samples grown at 50 and 30 sccm reveal distinct Mo_2_C peaks, absent in samples synthesized at higher sulfur flow rates. This is attributed to carbon from the precursor being trapped within the Mo film by horizontally oriented MoS_2_ layers, which act as an effective diffusion barrier. In contrast, the random layers formed under high sulfur flux provide multiple diffusion pathways, allowing carbon to escape rather than crystallize into molybdenum carbide. These observations suggest that horizontally oriented films grown at low sulfur flux form more uniform and efficient barrier layers than the partially random layers generated at high flow rates. The persistent Mo peaks in all XRD patterns confirm the intentional retention of the metallic substrate, enabling orientation studies on practical metallic surfaces that serve as both growth templates and electrical contacts.

Analysis of the full width at half maximum (FWHM) of the *A*_1g_ peaks (Figure 2d) reveals that samples grown at low sulfur flow rates (30 and 50 sccm) exhibit narrower FWHM values (Δω = 4.2 ± 0.3 cm^−1^) compared to those grown at high flow rates (Δω = 7.8 ± 0.5 cm^−1^ at 1000 sccm). To distinguish whether this broadening reflects crystalline disorder or orientation mixing, we performed XRD Scherrer analysis on well-defined peaks across all samples (Supplementary Table S2). The results show constant peak widths—MoS_2_ (002) peaks consistently 0.38–0.42° and MoS_2_ (110) peaks consistently 0.58–0.64°—corresponding to crystallite sizes of 20–25 nm independent of sulfur flux. This constancy of peak width despite dramatic orientation changes (Figure 2b) rules out crystalline disorder. Attempted two-component deconvolution of the *A*_1g_ peak for the vertically dominant sample (Supplementary Figure S4) reveals spectral complexity consistent with the mixed-orientation character of the surface random layer. The FWHM broadening arises from the structural heterogeneity of this surface layer observed in TEM (Figure 3c,d and Figures S1–S3). This layer, consisting of collision zones between independently nucleated vertical domains, contains both near-vertical and tilted/horizontal-like lamellae (white arrows in Figures S1 and S2), as directly confirmed by high-resolution TEM imaging. Raman spectroscopy with 532 nm excitation (penetration depth ~50 nm) is particularly sensitive to this surface region, whereas XRD reflects bulk vertical orientation throughout the film thickness. The systematic FWHM trends, validated by XRD Scherrer analysis (constant crystallite size from reliable peaks) and TEM morphology (orientation mixing), provide a robust descriptor of this surface/bulk structural complexity.

The orientation transition inferred from XRD and Raman analyses was further corroborated by cross-sectional TEM images of samples grown under sulfur flow rates of 30 and 1000 sccm (Figure 3). The sample synthesized at 30 sccm (Figure 3a,b) exhibits well-defined horizontally aligned layers with uniform interlayer spacing oriented parallel to the substrate surface. Despite minor substrate undulations, the MoS_2_ layers conform to the surface contours while preserving their horizontal alignment, consistent with the XRD results. In contrast, the sample grown at 1000 sccm (Figure 3c,d) displays distinct vertically aligned layers perpendicular to the substrate. The magnified view (Figure 3d) reveals the detailed structure of these vertical arrangements. Near the surface, the first several nanometers form a transitional layer with partially random orientation, which gradually evolves into the well-defined vertical alignment observed throughout the film thickness. Additional high-resolution TEM images of the random orientation layers are provided in the Supplementary Information (Figures S1–S3). These images reveal intriguing structural features, including competing growth directions, collision zones, and complex layer interactions. The random-layer regions contain intermixed horizontal and vertical segments, forming unique boundary structures that further elucidate the mechanisms underlying orientation control.

## 4. Discussion

### 4.1. Reaction-Diffusion Model Development

To elucidate the relationship between sulfur flux and MoS_2_ orientation, a reaction-diffusion model was developed, capturing the competition between surface reactions and diffusion processes during film growth. The fundamental reaction–diffusion equation describing sulfur transport and consumption during MoS_2_ formation is expressed as follows [45,57]:

(1) $$\partial C/\partial t=D\nabla^{2}C-r\left(F\right)\cdot C$$

where *C*(*x*,*y*,*z*,*t*) is the sulfur concentration mol/m^3^, *D* is the diffusion coefficient m^2^/s which effectively incorporates the complex, flux-dependent, and anisotropic diffusion behavior (*D_eff_*) detailed in the Supplementary Information (Section S3), and *r*(*F*) is the reaction rate constant *s*^−1^, which is a function of the supplied sulfur flux *F* sccm.

Under steady-state conditions and assuming predominantly vertical diffusion in layered structures, the equation can be simplified to the following:

(2) $$d^{2}C/dz^{2}=r\left(F\right)/D\cdot C$$

Therefore, the concentration profile of sulfur within the growing film can be expressed as follows:

(3) $$C\left.\left(z\right.\right)=C_{0}\left.\left(F\right.\right)e^{\left\{-z/\lambda \right\}}$$

where $C_{0}\left(F\right)=\alpha F\;mol/m^{3}$ is the surface concentration proportional to the supplied flux, and $\lambda =\sqrt{D/r\left(F\right)}$ m is the characteristic diffusion length.

(4) $$r\left(F\right)=r_{m}ax\times F/\left(F+K_{m}\right)$$

where *r_max_ s*^−1^ is the maximum reaction rate and *K_m_* sccm is the half-saturation constant. This formulation captures the transition from first-order kinetics at low flux (*r*(*F*) ∝ *F* when *F* << *K_m_*) to zero-order kinetics at high flux (*r*(*F*) ≈ *r_max_* when *F* >> *K_m_*). This transition occurs around *K_m_* = 100 sccm, consistent with experimental observations.

(5) $$D_{eff}\left(F\right)=D_{⊥}+\left(D_{∥}-D_{⊥}\right)\cdot F^{n}/\left(F^{n}+F_{d}^{n}\right)$$

where *D*_⊥_ *m*^2^/*s* and *D*_∥_ *m*^2^/*s* are the diffusion coefficients perpendicular and parallel to the layers, respectively, *F_d_* sccm is the diffusion transition threshold, and *n* controls the transition sharpness. This model considers the layered structure of MoS_2_, where diffusion parallel to the layers (through van der Waals gaps) is substantially faster than perpendicular diffusion across covalent bonds. The ratio *D*_∥_/*D*_⊥_ ≈ 100 reflects the two-order-of-magnitude difference in diffusion pathways, consistent with literature reports for layered materials.

**Figure 4:**
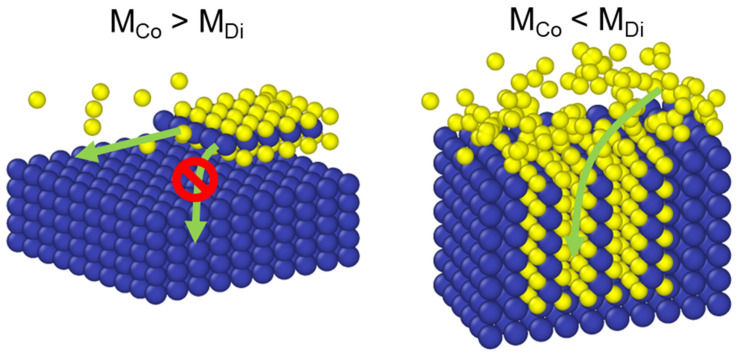
Schematics of the proposed MoS_2_ orientation-control mechanism based on the competition between the surface reaction (*M_Co_*) and diffusion (*M_Di_*) rates at different sulfur-flux conditions.

A key feature of the developed model is the introduction of the Thiele modulus (*φ*), a dimensionless parameter that quantifies the competition between reaction and diffusion, as follows:

(6) $$\varphi^{2}\;=\;L^{2}\;\times \;r(F)/D_{eff}\left(F\right)$$

where *L m* is the thickness of the characteristic film; this parameter effectively distinguishes between reaction-limited growth (corresponding to a large *φ*, which leads to horizontal-orientation growth) and diffusion-limited growth (corresponding to a small *φ*, which promotes vertical-orientation growth). The critical insight is that the Thiele modulus *φ* = *L/λ* quantifies the competition between reaction and diffusion. When *φ* >> 1 (reaction-limited), sulfur is consumed near the surface, promoting horizontal growth. When *φ* << 1 (diffusion-limited), sulfur penetrates deeply before reacting, enabling vertical growth. When *φ* ≈ 1, comparable rates of reaction and diffusion result in mixed orientation. Figure 4 conceptually illustrates this mechanism based on the competition between surface reaction (*M_Co_*) and diffusion (*M_Di_*) rates under different sulfur-flux conditions.

### 4.2. Modeling Orientation Components

To quantitatively capture the experimentally observed orientation transition, the relative contributions of different orientation components were modeled as functions of the sulfur flux. The model incorporates horizontal (*H*(*F*)), random (*R*(*F*)), and vertical (*V*(*F*)) orientation functions (see Supplementary Information, S4). These functions are normalized to determine their relative contributions at a given sulfur flux as follows:

The Horizontal Orientation Function:

(7) $$H\left(F\right)=1/\left(1+e^{\left(k_{H}\times \left(F-F_{H}\right)\right)}\right)$$

The Random Layer Function:

(8) $$F\;<\;F_{c}\;:\;R(F)\;=\;0$$

(9) $$F\geq F_{c}:\;R\left(F\right)=R_{max}\times {\left(F-F_{c}\right)}^{m}/\left(K^{m}+{\left(F-F_{c}\right)}^{m}\right)$$

The Vertical Orientation Function:

(10) $$V\left(F\right)=1/\left(1+e^{\left(-k_{V}\times \left(F-F_{V}\right)\right)}\right)$$

These functions are normalized to calculate their relative contributions at any given flux, as follows:

(11) $$H_{n}orm\left(F\right)=H\left(F\right)/\left(H\left(F\right)+R\left(F\right)+V\left(F\right)\right)$$

(12) $$R_{n}orm\left(F\right)=R\left(F\right)/\left(H\left(F\right)+R\left(F\right)+V\left(F\right)\right)$$

(13) $$V_{n}orm\left(F\right)=V\left(F\right)/\left(H\left(F\right)+R\left(F\right)+V\left(F\right)\right)$$

To directly compare the outcomes of the developed model with the experimental XRD measurements, the theoretical orientation index (*OI*) was defined as follows:

(14) $$OI\left(F\right)=H_{norm}\left(F\right)\cdot \left(+1\right)+R_{norm}\left(F\right)\cdot \left(0\right)+V_{norm}\left(F\right)\cdot \left(-1\right)$$

This formulation assigns +1, 0, and −1 to perfectly horizontal, random, and perfectly vertical growth, respectively. This theoretical index can be directly compared with the experimentally defined orientation parameter *H* = (*A* − *B*)/(*A* + *B*), where *A* and *B* represent the normalized intensity of the MoS_2_ (002) (horizontal orientation) and (110) (vertical orientation) peaks, respectively. In the proposed formulation, *H_norm_* corresponds to *A*/(*A* + *B*), and *V_norm_* corresponds to *B*/(*A* + *B*), making the two indices mathematically equivalent when the random component is minimal, as follows:

(15) $$H=\left(A-B\right)/\left(A+B\right)=\left(H_{n}orm-V_{n}orm\right)/\left(H_{n}orm+V_{n}orm\right)\approx OI\left(F\right)$$

**Figure 5:**
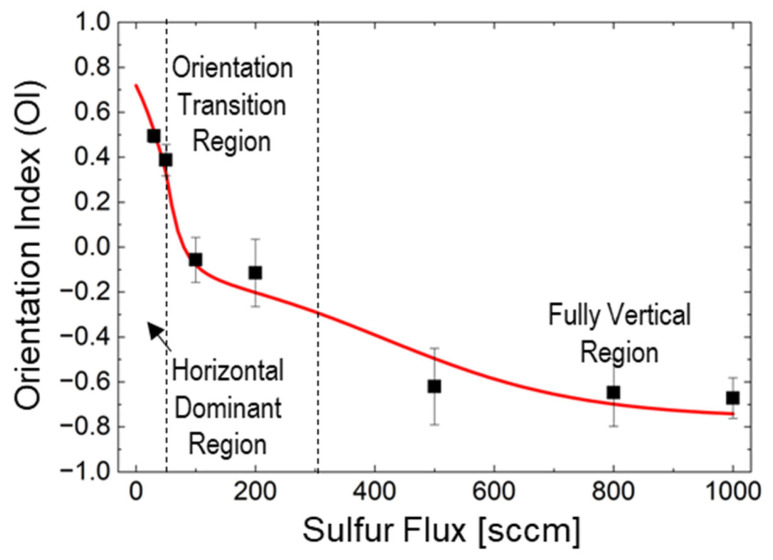
Plot of MoS_2_ orientation index values as a function of the sulfur flux to analyze the correlation between the model predictions and experimentally measured values.

When the random component is significant, the *OI* accounts for this additional contribution, thereby extending the experimental index to provide a comprehensive description of the system. Model predictions in Figure 5 were calculated using critical thresholds determined through a combination of experimental observations and model fitting. *F*_1_ = 50 sccm was identified from XRD analysis as the flux where the horizontal- to-mixed orientation transition begins, marked by the emergence of random layer contributions. *F*_2_ = 300 sccm, was derived through model optimization to match the experimental transition from mixed to vertical-dominant orientation observed between 200 and 500 sccm. The model parameters (*kH* = 0.018 sccm^−1^, *kV* = 0.006 sccm^−1^) are detailed in Supporting Information Table S1 and fitted parameters (*k_H_* = 0.018 *sccm*^−1^, *k_V_* = 0.006 *sccm*^−1^) detailed in Supporting Information (Table S1). The plot indicates three distinct growth regimes corresponding to different orientation behaviors. The critical thresholds (*F*_1_ = 50 *sccm*, *F*_2_ = 300 *sccm*, and *F_c_* = 30 *sccm*) marked with vertical dashed lines indicate excellent agreement between the model predictions (red line) and experimental data points (black squares).

The predictions of the proposed model are generally consistent with experimental observations across the full range of sulfur flux values. Agreement is particularly strong in the low-flux (<50 sccm) and high-flux (>500 sccm) regimes. Slight discrepancies arise in the transition region around 200 sccm, likely due to the complex dynamics during orientation switching, where local variations in sulfur concentration and diffusion pathways can significantly influence growth. By focusing deliberately on sulfur flux as the sole variable, this study isolates its effect and establishes a unified methodology for orientation control using identical substrates and processing conditions. This approach parallels the successful optimization of graphene growth on Cu substrates, which facilitated commercial-scale production. Importantly, the threshold transitions at ~50 sccm and ~300 sccm were reproducibly observed across multiple samples, and the reaction-diffusion model provides quantitative predictions that capture experimental trends within the precision limits of our characterization techniques.

The proposed model identifies two critical flux thresholds that govern the orientation transition:

‑ *F*_1_ ≈ 50 sccm: This threshold marks the transition from horizontal to mixed orientation, where *H*(*F*_1_) = *R*(*F*_1_).
‑ *F*_2_ ≈ 300 sccm: This threshold indicates the shift from mixed to vertical-dominated orientation, where *R*(*F*_2_) = *V*(*F*_2_).

The critical thresholds identified in this study—*F*_1_ ≈ 50 sccm and *F*_2_ ≈ 300 sccm—delineate three distinct growth regimes. While *F*_1_ was directly observed from experimental data, *F*_2_ represents the model-predicted center of the transition region experimentally observed between 200 and 500 sccm. The model’s ability to quantitatively capture this transition validates the reaction-diffusion framework and provides predictive capability for intermediate flux values not directly measured.

#### 4.2.1. Horizontal-Dominant Region (0–50 sccm)

In the low-flux regime, sulfur is primarily consumed at the surface, leading to the dominance of horizontal orientation (*OI* > 0). The Thiele modulus in this regime is relatively large (*φ* > 1), indicating that the reaction rate exceeds the diffusion rate. This generates a steep concentration gradient near the surface, promoting layer-by-layer growth parallel to the substrate. Experimentally, this is consistent with the intense MoS_2_ (002) peak and nearly undetectable (110) signal observed at 30 sccm (Figure 2a). The distinct Mo_2_C peaks observed at 30 and 50 sccm (Figure 1a) suggest that horizontally oriented MoS_2_ layers act as an effective diffusion barrier, trapping carbon from the precursor within the Mo film. This behavior aligns with previous reports by Jung et al., which indicate that significant volume expansion during sulfurization favors horizontal growth under limited diffusion conditions [45]. Additionally, the narrower FWHM values of the *A*_1g_ peaks for samples grown at low sulfur flow rates (30 sccm) further confirm a relatively uniform crystal structure in the absence of a significant random layer (Figure 2d).

#### 4.2.2. Transition Region (50–300 sccm)

As the sulfur flux increases to the intermediate range, excess sulfur begins to diffuse deeper into the film, promoting the formation of randomly oriented layers. In this regime, a gradual transition from horizontal to vertical orientation occurs, with the *OI* progressively shifting from positive to negative values. The Thiele modulus approaches unity (*φ* ≈ 1), indicating comparable rates of surface reaction and diffusion. This behavior aligns with the abrupt transition observed in the orientation parameter H within the 100–200 sccm range (Figure 2b), marking a critical threshold where growth kinetics shift from surface-reaction-limited to diffusion-limited. TEM images of the sample grown at 1000 sccm reveal an initial transitional layer with partially random orientation near the MoS_2_ surface (Figure 3c,d), supporting the inclusion of a random-layer function in the model. These observations are consistent with previous studies reporting the formation of an initial random layer during the early stages of vertical MoS_2_ growth [43,44]. Detailed analysis of these randomly oriented regions provides valuable insights into the mechanisms governing the horizontal-to-vertical orientation transition.

#### 4.2.3. Vertical-Dominant Region (>300 sccm)

In the high-flux regime, abundant sulfur diffusion drives predominantly vertical growth (*OI* ≈ −0.75). The Thiele modulus becomes small (*φ* < 1), indicating that diffusion rates exceed surface reaction rates. Under these conditions, sulfur penetrates deeply into the film before reacting, promoting growth perpendicular to the substrate. This is evidenced by the intense MoS_2_ (110) peak at 1000 sccm (Figure 2a) and the well-defined vertically aligned layers observed in TEM images (Figure 3c,d). Due to the anisotropic nature of layered materials, diffusion occurs more readily parallel to the layers (through van der Waals gaps) than across them, as suggested by Shang et al., highlighting diffusion kinetics as a key factor in MoS_2_ orientation [57]. This differential diffusion provides a kinetic advantage for vertical growth, allowing sulfur to penetrate along vertical channels and promote perpendicular layer formation. Raman FWHM analysis (Figure 2d) shows broader peaks for high-flux samples, attributable to the presence of a residual random orientation layer in the vertically aligned films, which contributes to vibrational-mode variations. Importantly, this random layer persists even at very high flux levels, explaining why the orientation index does not reach −1, consistent with TEM observations of residual random layers near the surface (Supplementary Figures S1–S3).

The deliberate focus on sulfur flux as the sole variable allows isolation of its effects and provides a unified methodology for orientation control using identical substrates and processing conditions. This approach parallels the successful development of graphene growth on Cu substrates, where process optimization enabled commercial viability. The 400 nm Mo thickness represents a sufficiently thick metallic substrate, where further increases would not affect surface growth dynamics, making it representative of practical metallic substrates for device applications. The underlying Mo layer serves as a conductive metallic substrate, analogous to how silicon wafers retain bulk Si beneath surface oxide layers. By employing partial sulfurization, this study enables the investigation of orientation control mechanisms on practical metallic substrates relevant for device integration. While the predictions of the proposed reaction-diffusion model align well with experimental data across the full sulfur flux range—particularly in the low-flux (<50 sccm) and high-flux (>500 sccm) regimes—slight discrepancies are observed in the intermediate transition region around 200 sccm. The primary focus of this work is to identify the conditions governing the extreme cases of horizontal and vertical orientation growth, rather than providing a detailed analysis of the transition regime. Future studies should aim to elucidate the precise mechanisms in this intermediate region, including the formation and evolution of random layers and their interactions with vertically oriented domains.

### 4.3. Role of Grain Boundaries in Diffusion Pathways

The polycrystalline nature of the sputtered Mo substrate introduces grain boundaries that could potentially serve as fast diffusion pathways for sulfur. However, several lines of evidence indicate that grain boundary diffusion does not dominate the orientation control mechanism described by the reaction- diffusion model: First, XRD analysis reveals that the Mo film exhibits relatively large grain sizes (dMo ≈ 45–50 nm, calculated from the Mo(110) peak width using the Scherrer equation), whereas the MoS_2_ films show significantly smaller grain sizes (dMoS_2_ ≈ 20–25 nm for both horizontal and vertical orientations). This substantial mismatch indicates that MoS_2_ nucleation and growth are not templated by Mo grain boundaries, suggesting that through-film diffusion rather than grain boundary diffusion governs the growth process. Second, cross-sectional TEM images (Figure 3) demonstrate that horizontally oriented MoS_2_ layers maintain their alignment even across visible grain boundaries in the underlying Mo substrate. If grain boundary diffusion were the dominant pathway, preferential vertical growth would be expected at these locations due to localized high sulfur concentrations. The observed continuous horizontal alignment across grain boundaries indicates that surface reaction kinetics—as captured by the Thiele modulus framework—control orientation rather than localized diffusion hotspots. Third, detailed examination of vertically oriented samples reveals complex boundary structures whose origins require careful interpretation. Supplementary Figures S1–S3 show the random orientation layer at the surface of the sample grown at 1000 sccm, where arrows indicate the growth directions of different MoS_2_ domains. These boundaries predominantly represent collision zones between independently nucleated vertical growth domains propagating in different azimuthal directions, rather than pre-existing Mo substrate grain boundaries. Several observations support this interpretation:

(i) The random orientation layer shows multiple growth fronts intersecting at various angles (as indicated by arrows in Figures S1–S3), with no systematic correlation to the underlying Mo grain structure visible in Figure 3c.
(ii) The stochastic distribution of these collision zones shows no preferential spacing or alignment that would correspond to the Mo grain size determined by XRD.
(iii) The growth directions (arrows) converge from multiple orientations toward collision points, consistent with surface nucleation followed by vertical propagation, rather than vertical growth originating from linear features (which would be expected if Mo grain boundaries were the primary nucleation sites).

It is important to note that distinguishing between growth-induced boundaries (resulting from domain collision) and diffusion-enhanced boundaries (resulting from preferential growth along pre-existing Mo grain boundaries) requires careful analysis. While the TEM images show numerous boundary structures, the lack of spatial correlation between these boundaries and the underlying Mo grain structure, combined with their random angular distribution, strongly suggests that they are primarily collision zones formed during growth rather than templated by substrate grain boundaries. While grain boundary diffusion undoubtedly contributes to the overall sulfur transport, particularly in the initial stages of growth, the effective diffusion coefficient *D_eff_* in the proposed model implicitly accounts for these contributions through its flux-dependent formulation Equation (5). The excellent agreement between model predictions and experimental data (Figure 5) validates this treatment, demonstrating that the competition between surface reaction and through-layer diffusion—as quantified by the Thiele modulus—remains the primary determinant of MoS_2_ orientation control.

### 4.4. Implications for Film Transfer and Device Integration

The retention of the Mo metal layer beneath the MoS_2_ film, as evidenced by persistent Mo peaks in the XRD patterns, provides a distinct advantage for practical applications. This metallic underlayer can be selectively removed using chemical etchants such as ammonium persulfate or ferric chloride, enabling the damage-free transfer of orientation-controlled MoS_2_ films onto arbitrary substrates. Such transfer capability is essential for integrating MoS_2_ with existing device architectures and offers a significant advantage over direct growth on insulating substrates, which limits flexibility in device design.

## 5. Conclusions

This study demonstrates precise control of MoS_2_ film growth orientation on non-noble metal substrates solely through sulfur flux regulation. The retention of the underlying Mo metal layer, confirmed by XRD analysis, is advantageous, as it enables potential film transfer via selective etching, analogous to the graphene/Cu system. The reaction-diffusion model based on the Thiele modulus provides quantitative predictions of orientation transitions, identifying critical thresholds at ~50 and ~300 sccm.

Experimental results from XRD, Raman spectroscopy, and TEM reveal a clear transition from horizontal-orientation growth at low sulfur fluxes (30–50 sccm) to vertical-orientation growth at high fluxes (>300 sccm). A reaction-diffusion model based on the Thiele modulus quantitatively captures this orientation transition and accurately identifies the critical sulfur-flux thresholds (~50 and ~300 sccm). This work represents the first demonstration of orientation control on thick metallic substrates using sulfur flux alone, providing a unified methodology comparable to the transformative growth strategies developed for graphene on Cu.

Despite the promising results, several limitations should be noted. While representative samples at each sulfur flow rate were characterized, the observed trends—including the threshold behavior at ~50 sccm and ~300 sccm—were reproducible across multiple growth runs; future studies with larger sample sizes would further strengthen statistical confidence. The proposed reaction-diffusion model simplifies the anisotropic diffusion behavior in polycrystalline Mo films and may not fully capture complex three-dimensional diffusion pathways. Functional characterization of the resulting MoS_2_ films is beyond the scope of this study; however, the demonstrated ability to selectively grow horizontal and vertical orientations provides a foundation for optimizing electronic (horizontal) and catalytic (vertical) applications, as reported in the literature. This selective growth of MoS_2_ orientations opens pathways for optimizing electronic (horizontal) and catalytic (vertical) applications as reported in the literature. The significance of this work lies in controlling different growth orientations on a single substrate through a unified process, enabling the integration of electronic and catalytic applications, which require horizontally and vertically grown MoS_2_, respectively. The growth of MoS_2_ by the proposed technique parallels the transformative development of graphene growth on Cu substrates and has the potential to accelerate the commercialization of MoS_2_-based technologies.

While complete sulfurization was not the objective—since the Mo substrate serves as both a growth template and a potential sacrificial layer for transfer—future work could focus on optimizing transfer processes and exploring epitaxial Mo substrates to enhance horizontal film quality. The methodology presented here establishes a foundation for the scalable production of orientation-controlled MoS_2_ films with transfer capability, thereby advancing the commercialization potential of TMDC-based technologies.

## Data Availability

The data supporting the findings of this study are available from the corresponding author upon reasonable request. Raw XRD, Raman, and TEM data, along with model parameters and calculations, are archived and available for research purposes in compliance with institutional data management policies.

## Supplementary Materials

The following supporting information can be downloaded at: https://www.mdpi.com/article/10.3390/nano15231783/s1, Supporting Information S1: Complete reaction-diffusion model derivation and parameter determination methodology; Supporting Information S2: Detailed Modeling of Sulfur Consumption Rate; Supporting Information S3: Anisotropic Diffusion and Barrier Effects; Supporting Information S4: Detailed Orientation Component Model; Supporting Information S5: Orientation Index Calculation and Parameter Determination; Supporting Information S6: Growth Regimes and Physical Interpretation; Supporting Information S7: Model Limitations and Future Work; Figure S1: Random orientation layers at the surface for the MoS_2_ grown in 1000 sccm of sulfur source; Figures S2 and S3: High-resolution TEM Images of the Random Orientation Layer; Figure S4: Two-Component Gaussian Deconvolution of Raman *A*_1g_ Peak for Vertically Dominant Sample; Table S1: Model Parameters and Sensitivity Analysis. Table S2: XRD Scherrer analysis demonstrating constant crystallite sizes across all flux conditions. References [42,58,59,60,61] are cited in the supplementary materials.
