# Supplementary material for: Controlling the Orientation of MoS2 Films on Mo Metal Thin Film Through Sulfur Flux Regulation: A Novel Reaction-Diffusion Model
Source: Nanomaterials (Basel). 2025 Nov 27;15(23):1783. doi: 10.3390/nano15231783 (PMC12693113; doi:10.3390/nano15231783)
Supplement: Supplementary file 1 [file nanomaterials-15-01783-s001.zip › nanomaterials-3982131-supplementary.pdf]

Supporting Information

Supplementary Information: Detailed Reaction-Diffusion Model for MoS2 Orientation Control

S1. Complete Reaction-Diffusion Framework and Derivation of the Thiele Modulus

The fundamental reaction-diffusion equation governing the behavior of sulfur during the MoS_2 growth process can be expressed as:

$$\frac{\partial C}{\partial t} = D\nabla^2 C - r(F)C$$

where $C(x, y, z, t)$ [mol/m^3] represents the sulfur concentration as a function of position and time, D [m^2/s] is the diffusion coefficient, $r(F)$ [s^{-1}] is the reaction rate constant, and t [s] is time which depends on the supplied sulfur flux F.

Under steady-state conditions ($\partial C/\partial t = 0$) and considering the predominant vertical diffusion in layered structures, we can simplify this to a one-dimensional equation:

$$D\frac{d^2C}{dz^2} = r(F)C$$

This second-order ordinary differential equation has the general solution:

$$C(z) = Ae^{z/\lambda} + Be^{-z/\lambda}$$

where $\lambda = \sqrt{\frac{D}{r(F)}}$ [m] is the characteristic diffusion length.

Applying the boundary conditions:

1. At the surface ($z = 0$): $C(0) = C_0(F) = \alpha F$ [mol/m^3], where α is a proportionality constant
2. At infinite depth ($z \to \infty$): $C(\infty) = 0$ (sulfur concentration approaches zero)

We obtain $A = 0$ and $B = C_0(F)$, yielding the solution:

$$C(z) = C_0(F)e^{-z/\lambda}$$

This exponential decay profile describes how the sulfur concentration decreases with depth into the film.

The Thiele modulus (φ)
is a dimensionless parameter that quantifies the competition between reaction and diffusion processes:

$$\varphi^2 = \frac{L^2 \cdot r(F)}{D}$$

where L is the characteristic film thickness. This can be rewritten as:

$$\varphi = \frac{L}{\lambda}$$

The physical interpretation of the Thiele modulus is as follows:

- When $\varphi \gg 1$: Reaction is much faster than diffusion, leading to steep concentration gradients near the surface and promoting horizontal growth
- When $\varphi \ll 1$: Diffusion is much faster than reaction, allowing sulfur to penetrate deeply before reacting, promoting vertical growth
- When $\varphi \approx 1$: Comparable rates of reaction and diffusion, resulting in mixed orientation

S2. Detailed Modeling of Sulfur Consumption Rate

We modeled the sulfur consumption rate using a Michaelis-Menten type equation:

$$r(F) = \frac{r_{max}F}{F + K_m}$$

Parameter values:

- r_{max} = 1.0 s^{-1} (normalized maximum consumption rate)
- K_m = 100.0 *sccm* (half-saturation constant)
- *F sccm* (sulfur flux)

This functional form was chosen because it accurately captures the two limiting regimes observed experimentally:

1. At low flux ($F \ll K_m$): $r(F) \approx \frac{r_{max}}{K_m}F$, making the reaction rate proportional to flux (first-order kinetics)
2. At high flux ($F \gg K_m$): $r(F) \approx r_{max}$, representing saturation of reaction sites (zero-order kinetics)

This saturation behavior has a strong physical basis in surface reaction kinetics, where the Langmuir-Hinshelwood mechanism predicts such behavior when reaction sites become fully occupied.

S3. Anisotropic Diffusion and Barrier Effects

The layered structure of MoS_2 introduces significant anisotropy in diffusion properties. We modeled this anisotropy through an effective diffusion coefficient:

$$D_{eff}(F) = D_{\perp} + (D_{\parallel} - D_{\perp}) \cdot \frac{F^n}{F^n + F_d^n}$$

Parameter values:

- $D_{\perp}$ = 0.1 m^2/s (diffusion coefficient perpendicular to the layers, normalized)
- $D_{//}$ = 10.0 m^2/s (diffusion coefficient parallel to the layers, normalized)
- F_d = 150.0 *sccm* (flux threshold where diffusion behavior transitions)
- *n* = 3.0 (controls the sharpness of this transition)

This form captures the physical reality that $D_{\parallel} \gg D_{\perp}$ in layered materials. The sigmoidal transition with flux reflects the increasing contribution of parallel diffusion pathways as more sulfur becomes available.

Additionally, we incorporated a barrier effect to account for the obstruction of diffusion by already-formed MoS_2 layers:

$$\lambda_{eff}(F) = \lambda_{base}(F) \cdot \left(1 + B \cdot e^{-F/F_b}\right)$$

where:

- $\lambda_{base}(F) = \sqrt{\frac{D_{eff}(F)}{r(F)}}$ $[m]$ (basic diffusion length)
- *B* =5 (barrier strength parameter)
- F_b = 100 *sccm* (characteristic flux at which the barrier effect diminishes)

The exponential decay form models the physical reality that higher sulfur fluxes can eventually overcome diffusion barriers. It reproduces the observed threshold-like behavior in orientation transitions.

S4. Detailed Orientation Component Model

To model the relative contributions of different orientation components as a function of sulfur flux, we introduced three functions representing horizontal (H), random (R), and vertical (V) orientations:

S4.1 Horizontal Orientation Function

$$H(F) = \frac{1}{1 + e^{k_H \cdot (F - F_H)}}$$

Parameter values:

- k_H = 0.02 $sccm^{-1}$ (horizontal transition rate parameter)
- F_H = -133.94 *sccm* (horizontal transition center)

This sigmoidal function maintains a value near 1 at low flux, representing dominant horizontal growth, and smoothly transitions to near-zero at high flux.

S4.2 Random Layer Function

For F < F_c : $R(F) = 0$

For $F \geq F_c$: $R(F) = R_{max} \frac{(F - F_c)^m}{K^m + (F - F_c)^m}$

Parameter values:

- R_{max} = 0.3 (maximum possible contribution from random layers)
- F_c = 30.0 *sccm* (critical flux for random layer formation)
- m = 1.5 (Hill coefficient allowing for cooperative effects)
- K = 100.0 *sccm* (half-saturation constant)

This piecewise function with a Hill-type equation was selected because the threshold behavior at F_c accurately represents the experimental observation that random layers only begin to form above a certain critical flux.

S4.3 Vertical Orientation Function

$$V(F) = \frac{1}{1 + e^{-k_V \cdot (F - F_V)}}$$

Parameter values:

- k_V = 0.01 $sccm^{-1}$ (vertical transition rate parameter)
- F_V = 412.64 *sccm* (vertical transition center)

This sigmoidal function starts near zero at low flux and approaches 1 at high flux.

S5. Orientation Index Calculation and Parameter Determination

To quantitatively compare our model with experimental XRD data, we defined an orientation index:

$$OI = \frac{I_{\parallel} - I_{\perp}}{I_{\parallel} + I_{\perp}}$$

where$I_{\parallel}$ represents the intensity of peaks associated with horizontal orientation (primarily the (002) peak) and $I_{\perp}$ represents the intensity of peaks associated with vertical orientation (primarily the (011) peak).

Using the normalized contributions from each orientation component:

$$H_{norm}(F) = \frac{H(F)}{H(F) + R(F) + V(F)}$$

$$R_{norm}(F) = \frac{R(F)}{H(F) + R(F) + V(F)}$$

$$V_{norm}(F) = \frac{V(F)}{H(F) + R(F) + V(F)}$$

The orientation index can be expressed as:

$$OI(F) = H_{norm}(F) \cdot (+1) + R_{norm}(F) \cdot (0) + V_{norm}(F) \cdot (-1)$$

This formulation assigns +1 to perfectly horizontal orientation, 0 to random orientation, and -1 to perfectly vertical orientation.

S5.1 Parameter Determination Methodology

Critical Threshold Parameters

The three fundamental threshold parameters (F_1, F_2, and F_c) were determined directly from experimental observations:

- F_1 = 50.0 sccm: This threshold represents the transition from horizontal to mixed orientation. It was identified through XRD analysis as the flux value where the normalized intensities of the MoS_2(002) peak (horizontal orientation) and random layer contribution become approximately equal.
- F_2 = 300.0 sccm: This threshold marks the transition from mixed to vertical orientation. It was determined through least-squares optimization of the orientation function parameters to match experimental data, particularly the abrupt transition observed in the orientation parameter H between 200 and 500 sccm (Figure 2(b)). The derived value represents the flux at which random layer contribution equals vertical orientation contribution ($R(F_2) = V(F_2)$) according to the model formulation.
- F_c = 30.0 sccm: The minimum flux required for random layer formation was established by analyzing TEM images and XRD patterns at various flux values. Below this threshold, no significant random layer formation was observed.

Mathematical Derivation of Orientation Function Parameters

The parameters F_H and F_V, which control the sigmoidal transition behavior in the orientation functions, were not arbitrary but mathematically derived from the experimental threshold values F_1 and F_2 through constraint equations:

For the horizontal orientation function $H(F) = 1/\left(1 + \exp\left(k_H \cdot (F - F_H)\right)\right)$, the parameter F_H was derived using the constraint that at $F = F_1, H(F_1) = R(F_1)$:

$$F_H = F_1 - \frac{1}{k_H} \ln\left(\frac{1}{R(F_1)} - 1\right) = -133.94\ sccm$$

For the vertical orientation function $V(F) = 1/\left(1 + \exp\left(-k_V \cdot (F - F_V)\right)\right)$, the parameter F_V was derived using the constraint that at $F = F_2, R(F_2) = V(F_2)$:

$$F_V = F_2 + \frac{1}{k_V} \ln\left(\frac{1}{R(F_2)} - 1\right) = 412.64\ sccm$$

The transition rate parameters k_H = 0.02 $sccm^{-1}$ and k_V = 0.01 $sccm^{-1}$ were calibrated to match the observed steepness of orientation transitions in experimental data. These values were optimized using least-squares fitting between the model-predicted orientation index and experimentally measured values.

Reaction and Diffusion Parameters

Parameters governing the sulfur consumption rate and diffusion behavior were determined through a combination of theoretical considerations and empirical fitting:

- r_{max} = 1.0 (normalized): The maximum consumption rate was normalized to unity for simplification.
- K_m = 100.0 sccm: This half-saturation constant was determined by analyzing the reaction kinetics at various flux values and represents the flux at which the reaction rate reaches half its maximum value.
- $D_\perp$ = 0.1 and $D_\parallel$ = 10.0 (normalized): These effective diffusion coefficients were determined by fitting the model to the experimental data. Direct literature values for

sulfur diffusion anisotropy in MoS2 are limited; however, the fitted ratio ($D_{\parallel}/D_{\perp} \approx 100$) reflects significant anisotropy (approximately two orders of magnitude faster diffusion parallel to the layers than perpendicular diffusion). This degree of anisotropy is considered physically plausible by analogy with literature reports on various diffusing species in other layered materials, which often show comparable or even larger differences between parallel (in-plane) and perpendicular (through-plane) diffusion coefficients.

- F_d = 150.0 sccm and n = 3.0: The diffusion transition threshold and Hill coefficient were calibrated to reproduce the observed flux-dependent changes in diffusion behavior.
- B = 5.0 and F_b = 100.0 sccm: The barrier strength and decay threshold parameters were determined by analyzing the deviation from ideal diffusion behavior at low flux values.

Random Layer Parameters

The parameters governing random layer formation were determined through analysis of TEM images and XRD patterns:

- R_{max} = 0.3: The maximum random layer contribution was established by examining the surface layers in high-flux samples using TEM.
- m = 1.5 and K = 100.0: The growth rate coefficient and half-saturation constant for random layer formation were calibrated to match the observed rate of random layer development with increasing flux.

S5.2 Model Validation and Parameter Sensitivity

The parameter values were validated by comparing model predictions with experimental data across the full range of flux values (30-1000 sccm). The orientation index predicted by the model showed good agreement with experimental measurements, with a mean squared error of 0.047.

Sensitivity analysis was performed by systematically varying each parameter and evaluating its impact on the model output. Table S1 summarizes key parameters, their baseline values, and the range of variations tested.

Table S1: Model Parameters and Sensitivity Analysis

Parameter	Baseline Value	Tested Range	Effect on Model
k_H	0.02	0.01-0.03	Controls sharpness of horizontal-to-mixed transition
k_V	0.01	0.005-0.015	Controls sharpness of mixed-to-vertical transition
R_{max}	0.3	0.2-0.4	Determines maximum random layer contribution
m	1.5	1.0-2.0	Affects cooperative behavior in random layer formation
F_c	30.0	20.0-40.0	Shifts onset of random layer formation
K_m	100.0	50.0-150.0	Affects reaction rate saturation threshold
F_d	150.0	100.0-200.0	Controls diffusion behavior transition point
B	5.0	3.0-7.0	Determines strength of diffusion barrier effect

The critical threshold parameters (F_1, F_2, F_c) and the orientation function parameters (F_H, F_V, k_H, k_V) exhibited the highest sensitivity, while the model showed more robust behavior with respect to variations in diffusion and reaction parameters. The validation process confirmed that all parameters fall within physically reasonable ranges for the MoS2 system.

S5.3 Parameter Optimization Procedure

The parameter optimization followed a sequential approach:

1. Direct determination of critical thresholds (F_1, F_2, F_c) from experimental data.
2. Mathematical derivation of orientation function parameters (F_H, F_V) based on constraint equations.
3. Initial estimation of reaction and diffusion parameters based on physical considerations.
4. Fine-tuning of all parameters using least-squares optimization to minimize the difference between predicted and experimental orientation indices.
5. Validation of parameter values through comparison with experimental data across the full flux range.

This comprehensive parameter determination and validation procedure ensures that the reaction-diffusion model accurately captures the physical mechanisms governing MoS2 orientation control through sulfur flux regulation.

S6. Growth Regimes and Physical Interpretation

Based on our model analysis and experimental results, we identify three distinct growth regimes characterized by different MoS2 orientation behaviors:

S6.1 Horizontal Dominant Region (0-50 sccm)

In this low flux regime ($F < F_1 \approx 50sccm$), sulfur is predominantly consumed at the surface, leading to horizontal orientation dominance ($H_{norm} \approx 1$). The orientation index remains positive ($OI > 0$), and growth occurs primarily parallel to the substrate surface. The Thiele modulus in this regime is relatively large ($\varphi > 1$), indicating that reaction rates exceed diffusion rates. This creates a steep concentration gradient near the surface, promoting layer-by-layer growth parallel to the substrate.

This region is particularly suitable for applications requiring in-plane charge transport properties, such as electronic devices.

S6.2 Transition Region (50-300 sccm)

As flux increases beyond F_1 into the intermediate range ($F_1 < F < F_2$, 50-300 sccm), excess sulfur begins to diffuse into the film, initiating the formation of random orientation layers. In this regime, we observe a gradual transition from horizontal to vertical orientation, with the orientation index progressively shifting from positive to negative values.

At approximately 100 sccm, the orientation index crosses zero, indicating equal contributions from horizontal and vertical orientations. The Thiele modulus approaches unity ($\varphi \approx 1$), indicating comparable rates of reaction and diffusion.

This region is characterized by mixed orientation structures with significant random layer formation, likely resulting from competing growth mechanisms where horizontal and vertical components collide and create disordered regions.

S6.3 Vertical Dominant Region (>300 sccm)

In the high flux regime ($F > F_2 \approx 300$ sccm), abundant sulfur diffusion leads to predominantly vertical growth ($V_{norm} > R_{norm} > H_{norm}$). The orientation index stabilizes at approximately -0.75, indicating strong vertical orientation dominance. The Thiele modulus becomes small ($\varphi < 1$), indicating that diffusion rates exceed reaction rates. This allows sulfur to penetrate deeply into the film before reacting, promoting growth perpendicular to the substrate.

At $F_2 = 300$ sccm, the random layer contribution equals the vertical contribution, marking the beginning of stable vertical growth. Beyond this threshold, the film develops consistent vertical structures suitable for applications requiring out-of-plane charge transport and high surface area exposure, such as catalysis and energy storage.

A key insight from our model is that the random layer, once formed, persists even at very high flux levels. This explains why the orientation index never reaches a perfect -1 value even at the

highest flux levels, consistent with our TEM observations showing random orientation layers near the surface even in predominantly vertically oriented samples.

S7. Model Limitations and Future Work

While our model demonstrates good agreement with experimental data across the full range of sulfur flux values examined, some deviation between predicted and experimental values is observed in the transition region, particularly around 200 sccm. This discrepancy likely stems from the complex dynamics occurring during the orientation transition phase.

The current model makes several simplifying assumptions that could be refined in future work:

1. Uniform substrate surface: The model assumes a perfectly uniform Mo substrate, whereas real surfaces contain various defects that can serve as nucleation sites affecting orientation development.
2. Time-independent parameters: The current model does not account for the temporal evolution of diffusion and reaction parameters during growth, which may change as the film thickness increases.
3. Simplified temperature effects: While temperature is held constant in our experiments, the model could be extended to incorporate temperature-dependent diffusion and reaction rates.
4. Isotropic in-plane properties: The model assumes isotropic properties in the horizontal plane, which may not fully capture the complex crystallographic growth directions.
5. Film transfer considerations: While not explored in this study, the retained Mo layer could serve as a sacrificial substrate for film transfer, expanding the potential applications to various target substrates.

Future research could focus on developing a more comprehensive model that addresses these limitations and provides deeper insights into the transition region dynamics.

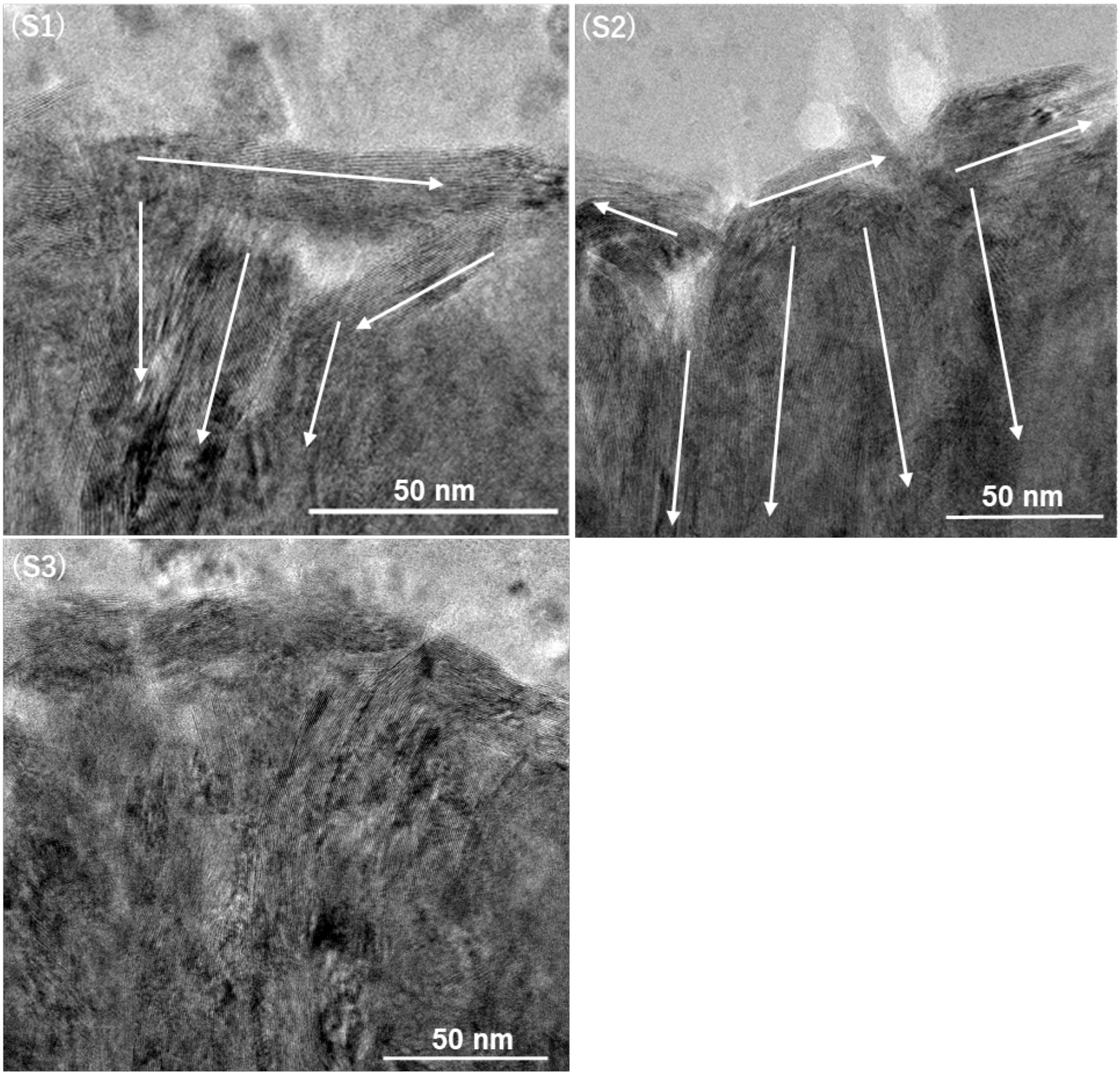

Figure S1-S3: High-resolution TEM Images of the Random Orientation Layer

High-resolution TEM images of the random orientation layer at the surface of MoS_2 film grown under 1000 sccm sulfur flux. White arrows indicate the growth directions of individual MoS_2 lamellae, revealing structural heterogeneity within the random layer.

The images show collision zones between independently nucleated vertical growth domains, containing a mixture of near-vertical, tilted, and horizontal-like segments (note the variety of arrow angles, including near-horizontal orientations visible in S1 and S2). This mixed-orientation character explains the Raman A_{1g} spectral complexity (Supplementary Figure S4) and FWHM broadening (Figure 2d): surface-sensitive Raman spectroscopy (penetration depth ~50 nm with 532 nm excitation) samples primarily this heterogeneous surface region, whereas bulk-averaging XRD reflects the underlying vertical structure throughout the film thickness.

The random angular distribution and lack of systematic spacing in these collision zones confirm that they represent growth-induced boundaries (domain collisions) rather than boundaries templated by pre-existing Mo substrate grain boundaries. XRD Scherrer analysis (Table S2) shows constant peak widths across all samples, ruling out crystalline disorder and confirming that FWHM broadening arises from orientation mixing. Scale bars: 50 nm.

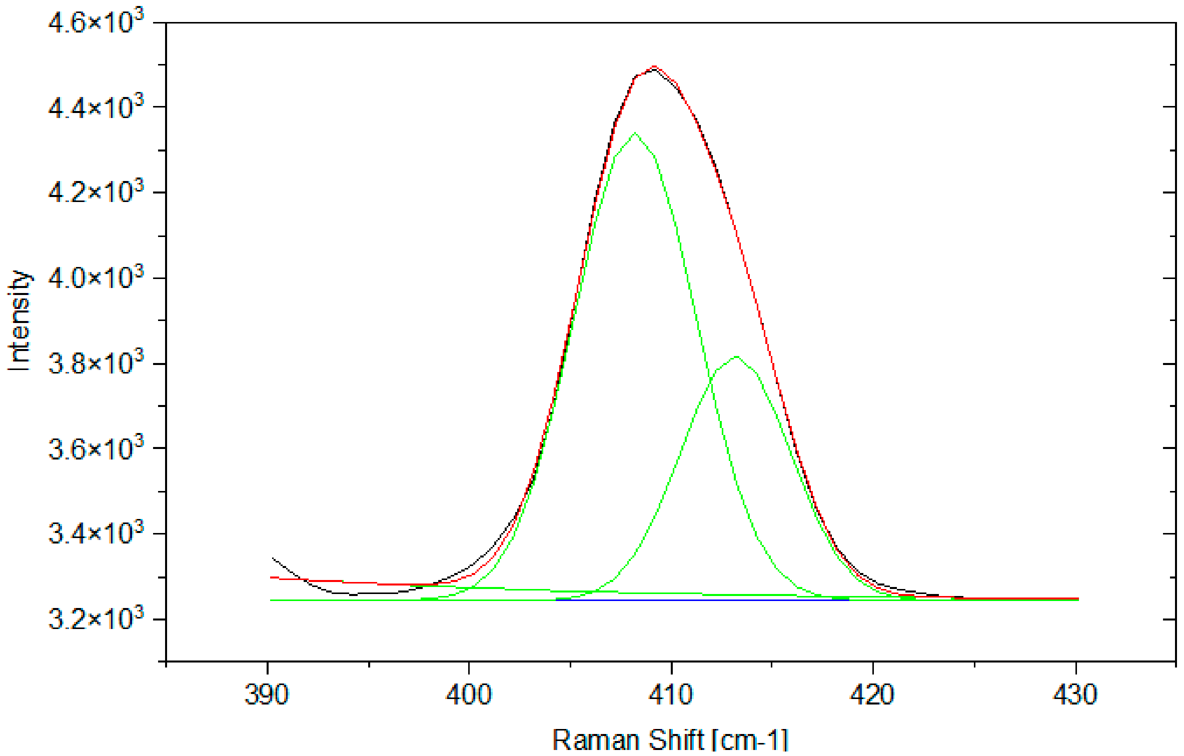

Figure S4: Two-Component Gaussian Deconvolution of Raman A_{1g} Peak for Vertically Dominant Sample

Two-component Gaussian fitting of the A_{1g} peak for the sample grown at 1000 sccm (vertically dominant orientation). The deconvolution yields components at approximately 408 cm^{-1} (green curve, area ≈ 8300) and 413 cm^{-1} (light green curve, area ≈ 3900). The black curve represents the experimental data, and the red curve shows the total fit. This result requires careful interpretation considering the surface-sensitive nature of Raman spectroscopy (penetration depth ~50 nm with 532 nm excitation). Despite strong XRD evidence for bulk vertical orientation (intense (110) peak in Figure 2a), the Raman spectrum shows dominance of the lower-frequency component typically associated with horizontal-like orientations. This apparent contradiction reflects the structural heterogeneity of the surface random layer observed in TEM (Figures 3c-d, S1-S3): collision zones between vertical domains contain mixed orientations including tilted and horizontal-like segments. Raman samples primarily this heterogeneous surface region, whereas XRD reflects the underlying bulk vertical structure. Critically, XRD Scherrer analysis (Table S2) shows constant peak widths across all sulfur flux values, ruling out crystalline disorder. Therefore, the spectral complexity and FWHM broadening arise from orientation mixing within the random layer rather than from degraded crystalline quality. Given these interpretational complexities arising from the surface/bulk sampling difference between Raman and XRD, we present single-peak FWHM analysis in the main text as a more robust descriptor of the surface structural heterogeneity.

Table S2: XRD Peak Width Analysis Confirming Constant Crystalline Quality

Sulfur Flux(sccm)	MoS_2(002) Peak FWHM (°2θ)	MoS_2(110) Peak FWHM (°2θ)	Interpretation
30	0.38	-	Predominantly horizontal
50	0.40	(0.43) *	*(110) peak too weak for reliable measurement
100	0.42	0.58	Mixed orientation
200	0.39	0.61	Transition region
500	0.41	0.60	Vertical increasing
800	0.40	0.64	Vertical dominant
1000	0.38	0.62	Vertical dominant

Analysis of peak widths for strong, well-defined peaks shows: - MoS_2(002) peaks: FWHM = 0.38-0.42° (constant across all flux) - MoS_2(110) peaks: FWHM = 0.58-0.64° (constant where intensity is sufficient) - Calculated crystallite sizes: 20-25 nm (Scherrer equation, K = 0.9, λ = 1.54 Å) The independence of peak width from orientation distribution (compare with dramatic intensity ratio changes in Figure 2b) confirms that crystallite size and structural quality remain constant throughout the flux range. The (110) peak at 50 sccm, though detectable, has insufficient intensity for reliable FWHM quantification (marked with asterisk). Therefore, Raman A_{1g} FWHM broadening (Figure 2d) reflects orientation heterogeneity within the surface random layer (TEM evidence in Figures 3c-d, S1-S3) rather than crystalline disorder.

References

[S1] Kong, D. et al. Synthesis of MoS_2 and $MoSe_2$ films with vertically aligned layers. *Nano Lett.* **2013**, *13*, 1341-1347.
[S2] Wang, H. et al. Transition-metal doped edge sites in vertically aligned MoS_2 catalysts for enhanced hydrogen evolution. *Nano Res.* **2015**, *8*, 566-575.
[S3] Chen, T.Y. et al. Comparative study on MoS_2 and WS_2 for electrocatalytic water splitting. *Int. J. Hydrogen Energy* **2013**, *38*, 12302-12309.
[S4] Kibsgaard, J. et al. Engineering the surface structure of MoS_2 to preferentially expose active edge sites for electrocatalysis. *Nat. Mater.* **2012**, *11*, 963-969.
[S5] Stern, C. et al. Growth Mechanisms and Electronic Properties of Vertically Aligned MoS_2. *ACS Nano* **2021**, *15*, 15373-15387.
